# "The Hospital" Nursing Mirror

**Published:** 1897-03-27

**Authors:** 


### "The Hospital" Nursing Mirror.

Being the Nursing Section of "The Hospital."

[Contributions for this Section of "The Hospital" should be addressed to the Editor, The Hospital, 28 & 29, Southampton Street, Strand, London, W.C., and should have the word "Nursing" plainly written in left-hand top corner of the envelope.]

### News from the Nursing World.

### ST. HELENA'S HOME OF REST.

H.R.H. Princess Christian was present on the 16th inst. at a concert at Bridgewater House in aid of the St. Helena House and Home of Rest for Friendless Girls at Clapham. This home was started two years ago by the Princess, and is a small convalescent home for girls discharged from the lock wards of hospitals There are twelve beds, which have been full ever since the establishment of the home. The matron is a trained, experience nurse, and has a fully-trained nurse under her. H.R.H. Princess Christian, the Countess of Arran, Mrs. Wyndham Baring (secretary), Mrs. Somerville Clerk, and Mrs. John Thynne are on the committee. The home has done excellent work, and most cheering results are reported.

### THE QUEEN'S COMMEMORATION FUND IN DUBLIN.

Some very satisfactory subscriptions were announced at the largely attended meeting recently called by the Right Hon. Ion Trant Hamilton, Lord Lieutenant of the county of Dublin, to inaugurate a fund for the Queen's Jubilee Nurses in Ireland. It is to this fund that Lord Iveagh has contributed his splendid donation of £12,500, and the Lord Lieutenant, Lord Ardilaun, and Sir Henry Cochrane have each given £500. The Archbishop of Dublin has sent £100, remarking that were it not for other claims he would gladly "give a hundred-fold," and many other handsome subscriptions have been received.

### NURSING AT MIDDLESEX HOSPITAL.

The annual report of the Middlesex Hospital notes amongst changes in the nursing staff the retirement during the past year of Miss Ashton, for many years sister of Forbes' Ward. Miss Ashton, on giving up her work, was granted the pension "to which her long service entitles her." Her place has been taken by Miss Edwards, formerly senior nurse of the ward. The health of the nurses throughout the year is stated to have been good, "and the general high standard of efficiency maintained." The lectures to the nursing staff, formerly given by Mr. Pearce Gould, have been undertaken by Mr. John Murray.

### A GRATEFUL CONSTABULARY.

Sister Fahey, sister-in-charge of the Constabulary Ward of Dr. Steeven's Hospital, Dublin, was the recipient of a very pleasant token of gratitude and appreciation the other day, when the members of the Force presented her with a gold brooch, fashioned after the pattern of the constabulary crest, with harp, crown, and shamrock. The gift was accompanied by a letter expressing the givers' recognition of Sister Fahey's devoted work on their behalf, and was presented through Miss Kelly, the lady superintendent of the hospital.

### WELLS NURSING INSTITUTION.

The annual general meeting of the Wells Nursing Institution was held on March 9th, under the presidency of the Rev. Prebendary Beresford. The report presented showed a considerable increase in the work, and testified to the "energy, activity, and diligence" of Nurse Smith. Three hundred patients were visited by her during the year, an increase of 74 over the number for the preceding year, and the visits reached a total of 4,748. Mrs. Algernon Crosse was re-elected secretary, and Mr. Edward Foster treasurer.

### QUEEN'S NURSES AT HAWICK.

The fourth general meeting in connection with the Hawick branch of the Queen's Jubilee Institute for Nurses was held in the Town Hall on February 11th, when a most gratifying and satisfactory report was read. Nurse Biggart is doing a good work in the town, the number of visits paid during the past year being 3,635. The funds are in a prosperous state, amply proving that the work is meeting with due recognition as a great benefit to the community.

### QUEEN'S NURSES AT WORTHING.

The annual meeting of the Worthing District Nursing Association, affiliated to the Q.V.J.I., was held early this month, when a satisfactory report was presented. The nurses work not only in Worthing itself, but in Heene, Tarring, and Broadwater, and the Chairman (Mr. Hargood) was certainly right in appealing for increased support from the clergy of the various parishes, to the sick poor of which the services rendered by the nurses are invaluable. We hope the Nursing Association will be remembered in this year's commemoration projects.

### NURSES IN COUNCIL.

The pleasant and wholly informal discussions on nursing questions which are being held each month at the Trained Nurses' Club, Buckingham Street, enable nurses to meet together and compare with mutual advantage their views and experiences on many matters. The much-vexed question of "The Private Nurse" held the field last Friday evening, in continuation of a former discussion. Several helpful criticisms and suggestions were put forward, the unanimous opinion being that the present system of hospital training needed supplementing in some way for those who intended taking up private nursing if they were to be fully equipped for the pitfalls and difficulties of that branch of their work.

### SENTIMENT VERSUS CHARITY.

The people of Lancaster have rejected a proposal to commemorate the present year by increasing the number of district nurses in the town, and have decided instead to spend £500 in erecting a statue of the Queen. Lancaster has, according to Councillor Satterthwaite (who advocated the nursing scheme), a population of 37,000, and boasts but one district nurse, who is supported by a ladies' society. There can be little doubt as to which of these proposals would best suit Her Majesty's own views, and many of her loyal subjects in Lancaster will regret that this matter has thus been settled.

### A DISGRACE TO WARWICK.

BEFORE passing resolutions "in favour of applying for a loan for £3,000 to provide new tramp wards," it would be well that the Guardians of Warwick paid some sort of attention to the grievances of their infirmary nurses. It is reported in the local press that at a recent meeting of the board, Mrs. James (Kenilworth) "commented upon the frequent change of nurses, and said that all the nurses who came there made the same complaint. The fact was that the wounds of the infirmary patients had to be dressed in the room in which the nurses had their meals. If such a state of things existed in a private house the sanitary authorities would soon move in the matter." It can only be described as a scandalous state of things, which ought not to be allowed to continue for one day longer, and we trust that the infirmary committee, who have been instructed to suggest some plan of dealing with it, will speedily evolve efficient means of improvement. Twelve pounds, the sum which they appear to propose spending on the "defective accommodation," is scarcely likely to effect much.

### THE NURSES' CO-OPERATION.

THE annual report of this successful society has just been issued, and is a most satisfactory one from every point of view. The number of nurses has increased to 400, and even this large staff is at times found to be insufficient to meet all the applications. The gross receipts for 1896 amounted to £33,395 0s. 3d., an increase of £7,197 4s. 11d. over those for the previous year. Of this amount the sum of £30,892 2s. 9d. has been handed over to the nurses, as compared with £24,228 7s. 10d. in 1895. The income of the society, derived from a deduction of 7½ per cent. from the gross receipts, with £42 17s. 5d. received as "Home Fees," and £46 12s. 3d. interest on investments, amounted to £2,592 7s. 2d. Income exceeded expenditure by £1,006 12s. 9d., an increase of £182 10s. 5d., over the amount saved in the previous year. The capital of the co-operation now amounts to £3,067 2s. 8d., of which £2,400 is invested in Local Loans Stock. The total number of nurses admitted to the staff since the foundation of the society amounts to 663. The committee express great appreciation of Miss Philippa Hicks' "unflagging personal devotion," and their "unqualified approval of the excellent system on which her department is conducted."

### NURSES FOR INDIA.

THE six additional nurses asked for by the Government in India have now been selected by the India Office, and leave London on Friday for Bombay. They are Miss Franklin, of St. Thomas's Hospital; Miss Katsh, Miss Reynolds, and Miss Oram, of St. Bartholomew's Hospital; Miss Morey, of Hobart Town, Tasmania; and Miss Kendall, of the Adelaide Hospital, Dublin. They go out under the same terms as those of which we gave a *résumé* in THE HOSPITAL for the 13th inst. in regard to the first detachment.

### THE PLAISTOW MATERNITY CHARITY.

ANYBODY who knows Plaistow knows the vast amount of work which is accomplished amongst the poor of that dismal region by the Maternity Charity and District Nurses' Home, under Sister Katherine Twining's superintendence. A "Cake, Apron, and Pinafore Sale" is to be held at the end of May in aid of the charity, towards which contributions are much wanted. The Home urgently needs funds, both to help it to carry on its work, and to wipe off a debt of £500, and if plenty of "pinafores" are sent to Miss M. M. Birkett, 14, Chenies Street Chambers, Bloomsbury, or to Sister Katherine at the Home, Howard's Road, Plaistow, the sale should be a great success and contribute handsomely towards this end.

### THE VICTORIAN ERA EXHIBITION.

THE London Hospital has undertaken the supervision of the Nursing Section which is to have an important place in the Victorian Era Exhibition, to be opened at Earl's Court in May. Visitors to the East London Exhibition last year will remember that Miss Lückes undertook the charge of a "hospital stall" there, and that the particular corner, presided over by a London Hospital sister in her pretty blue uniform, was one of the great attractions of that excellent exhibition. The "Nursing Section" at Earl's Court is certain to be popular.

### IN THE LAW COURTS.

AN action for breach of promise of marriage was tried before Mr. Justice Hawkins and a special jury last week in which the plaintiff was Miss May Louise Maunder, a nurse at the South Devon and East Cornwall Hospital, Plymouth, and the defendant, Mr. William Hoare Brenton, a surgeon practising at Plymouth. The defendant pleaded that if he ever made the promise alleged he was induced to do so by the misrepresentations of the plaintiff respecting her social position. In cross-examination, the plaintiff denied that she had misled Dr. Brenton on this point. After Dr. Brenton's evidence had been taken, the jury handed a note to the judge saying that they did not think the defendant was exonerated from his promise, and they awarded the plaintiff £300 damages, for which judgment was accordingly entered.

### SHORT ITEMS.

MISS MASSON, late matron of the Radcliffe Infirmary, left her post last week. Miss Masson resigned owing to the retrograde policy adopted by those in authority, which rendered it impossible for her to maintain any standard of training amongst her nurses. Miss Masson left the infirmary, regretted by many, after years of hard work amidst the most discouraging circumstances. We heartily wish her happier experiences elsewhere.—Lady Pirbright has sent a hundred guineas to the Lady Mayoress's branch of the Prince of Wales' Hospital Fund, in memory of her father, Sir Benjamin Phillips, who was Lord Mayor of London in 1866 and 1867.—A paper on "Home Nursing" was read at the last meeting of the Manchester Ladies' Literary Club by Miss Wareing, of the Ramsbottom Hospital. It was specially intended to help ladies working amongst the poor.—Mrs. Stewart Brown has been admitted Lady of Grace of the Order of St. John of Jerusalem, an honour which has given much pleasure to that lady's many friends in Liverpool, where she has done so much for the poor and sick.—A successful dancing and gymnastic entertainment was given by Miss Harding and her pupils in St. Cuthbert's Hall, Edinburgh, the other day, in aid of the Royal Edinburgh Hospital for Sick Children.—The matinee at the Lyceum Theatre in aid of the Queen's Commemoration Fund on behalf of the Jubilee Institute for Nurses is announced for Tuesday, June 29th. Mrs. Keeley has promised to recite some lines specially written for the occasion, and Madame Sarah Bernhardt has also undertaken to give a recitation.—The annual meeting of the Hospital and Home for Incurable Children, 2, Maida Vale, was held at the Home on March 13th.

### Pharmacy and Dispensing for Nurses.

By C. J. S. THOMPSON.

### VI.—THE ART OF DISPENSING—THE PRESCRIPTION—WEIGHING AND MEASURING.

THE art of dispensing consists in the compounding of medical prescriptions in a proper and scientific manner. In order to become a competent and proficient dispenser, practical instruction and considerable experience are absolutely necessary, therefore it must not be expected from the outset, that an art which is by no means easy to acquire, can be mastered from a simple description of its various operations.

Dispensing presents no special difficulties which a woman, by study, practice, and careful attention, should not be able to overcome. Delicate manipulation and care are two very essential points in dealing with minute quantities such as grains and drops, and this carefulness in every detail should be cultivated from the beginning. Besides the accomplishment of technical and manipulative difficulties which can only be surmounted by experience, a knowledge of the structure and chemical composition of drugs and other medicinal agents, and the sciences of chemistry and botany which bear on the art, is necessary. A familiarity with the physical appearance of the drugs and chemicals used in medicine, with their preparations and their doses, is also of importance.

The position of the dispenser, on whose accuracy human life often depends, is one of great responsibility, and the compounding of medicine demands both scrupulous care and undivided attention. These important principles should ever be borne in mind.

When dispensing, the whole attention must be concentrated on the operation in hand, and not relaxed until it is completed. Once allow it to be distracted by conversation or otherwise, it is very easy to make an error which may have fatal results. The greatest care should be exercised over the smallest details, as, for instance, in draining the last drop of liquid from a measure, or in removing every particle of a pill mass from the mortar in which it has been made, and in noting that the measure or implement is perfectly clean before being used.

The strictest accuracy is also necessary in weighing and measuring where exactitude is of the utmost importance. Equal caution must also be observed in checking the doses ordered in prescriptions, and in the quantities used, also in writing the directions on the label.

Neatness is essential in every operation of dispensing, from the wrapping of a bottle or powder, to the rounding of a pill. The directions on the label should not only be written neatly, but distinctly, to prevent any possibility of error.

The practice of the rules we have mentioned should be habitual from the beginning, as when once a slovenly and careless method of work is acquired, it is rarely got rid of afterwards. It should be firmly resolved to use every precaution that will help to render a mistake a practical impossibility. One such error is sufficient to destroy confidence, and the unreliable dispenser can never be trusted.

### THE PRESCRIPTION.

The prescription is the medium of communication between the prescriber and the dispenser, wherein the former gives the ingredients and instructions for preparing and administering the medicinal agents to be dispensed. The word is derived from *præ* before, and *scribo* I write. In most countries Latin is used in writing medical prescriptions, for which purpose it has many advantages.

In the first place, it is a universal language among medical practitioners and pharmacists, and, therefore, a prescription written in Latin can be dispensed in almost any civilised country. Secondly, it is considered by prescribers not always advisable for the patient to be aware of the exact nature of the medicine he is taking. Therefore, to translate and read a prescription some knowledge of Latin is absolutely necessary.

The Latin names of the various drugs, chemicals, and their preparations must be committed to memory.

The names and words are largely abbreviated in practice, often to an extent that becomes confusing to the student, but with experience and practice this difficulty will soon be overcome. Thus tinctura is usually written as T. or Tr., extractum as E. or Ext., and liquidum as L. or Liq.

In the directions for administration also, the words used are largely abbreviated, but the terms generally employed are not very numerous, and may be learnt in a short time with a little careful study.

The prescription may be divided into four parts. (1) The heading. This usually begins with the name of the patient. Beneath it, in the left-hand corner, we have the symbol ℞, which represents the word "Recipe," signifying "Take thou." This symbol is said to have originated in the ancient invocation to Jupiter, with which the early physicians commenced their prescriptions. (2) The names of ingredients prescribed, each occupying one line, followed by the symbol denoting the weight or measure to be used. (3) The instructions to dispenser as to the form in which the medicine is to be made, as, for example, the mixture, the application, the pills, the powders, &c. (4) The directions for administration, the whole of which is to be translated and written in English on the label. Below, in the right hand corner, the initials or signature of the prescriber is usually placed, followed by the date on which the prescription was written.

The abbreviated manner in which some prescribers write the names of the ingredients in their prescriptions is often a source of difficulty to the student, especially when there is a similitude between the names of drugs. In such cases the dose ordered serves as a guide to the dispenser, but in cases where there is room for doubt the prescriber, if possible, should be communicated with. The following may be taken as examples of doubtful curtailment of words. Ext. col. might mean extract of colocynth or extract of colchicum, two very different preparations. Hydr. chlor. might be taken to mean calomel or corrosive sublimate, a mistake which might lead to very serious results, the latter being a very powerful poison. Potass. sulph. might be read as sulphate of potass or sulphurated potass, and many other instances of a similar nature might be enumerated against which the dispenser must be on guard.

As science has advanced, many changes in chemical nomenclature have been made, with the result that certain chemical and other bodies are known by various names. For instance, perchloride of mercury is also known as bichloride of mercury, corrosive sublimate, and mercuric chloride. Persulphate of mercury is also called sulphate of mercury, and mercuric sulphate, and sulphurated antimony is also known as oxy-sulphurat of antimony, golden sulphurat of antimony, and precipitated sulphurat of antimony. The dispenser should make a note of such synonyms until thoroughly acquainted with them.

In dispensing, as in the pharmacopœia, the rule that all liquids are to be measured and solids weighed, is strictly adhered to. In Great Britain apothecaries' weights and measures are employed in dispensing, but in continental countries and in the United States the metrical system is universally used. In Germany and France it is customary to weigh both solids and liquids in dispensing.

The weight or measured quantity of each ingredient in a prescription is expressed by the symbol which follows it; thus m. represents the minim, followed by the number in Roman numerals; gtt signifies drops, gr. for grain, ℈ the scruple, ʒ the drachm fluid or solid, ℥ the ounce, fluid or solid, ss following any of these symbols signifies half, for instance, ʒss represents half a drachm, and ℥iss one ounce and a half. The symbol O represents 1 pint or 20 fluid ounces, and C one gallon.

In weighing a powdered substance, a small quantity should first be placed in the scale pan and added to gradually by means of a palette knife, until the exact amount is reached to balance the scale. The same care should be exercised in measuring liquids. The measure should be raised to the level of the eye, and the fluid gently poured into it until the desired graduated mark is reached. Then it should be compared with the mark on the other side, taking care that the measure is held perfectly level.

### Post-Graduate Clinics for Nurses.

By a Trained Nurse.

### VII.—THE PERSONNEL OF THE NURSE.

Every text-book so far written has placed before us a separate and distinct ideal of the nurse as she might be. The newspapers and magazines are now helping to mould her character and correct her of her faults by showing these in no amiable light, and by dwelling on the virtues various contributors to the press consider a nurse can so easily develop. At one time, as an interesting study, I made a list of the excellences set forth in some dozen text-books as being essential to the private nurse. The list is before me as I write. From this it appears that no woman can aspire to the title of nurse unless she be prepared to relinquish many of the attributes common to humanity. She should need little sleep, and yet be invariably bright and cheerful. She must "cough gently" and never sneeze, her conversation should be "well-chosen and inspiriting," her countenance "open and well favoured," her mind refined, her body muscular. In full she must combine in her one person all the graces and virtues that go to make up a perfect human being. We were told recently in a magazine that a nurse ought to be a true Christian, humble, devoted, celibate, and religious. The same article also insisted that this ideal creature should take her meals in the kitchen. Now, it would appear to most of us if such a paragon of perfection entered our houses, we should either relegate her to a glass case, or regard her as so holy that we should not only proudly welcome her at our tables, but should consider it an honour to serve her on bended knee.

Perhaps this absolutely impossible ideal with regard to nurses which has been set up in the public mind is sufficient explanation of the disappointment we cannot deny many persons experience when first the necessity arises of admitting a trained nurse to their households. They expect more than a human woman can fulfil, for nature is more sparing of her gifts, and does not endow one mortal with perfections which would divide up generously among half a dozen. Personally, my experience of nurses in many countries and under very varying conditions has left me with the highest appreciation of them as a class. And perhaps the finest types of womanhood I have met have been in the nursing ranks—more especially among those of the "older-fashioned" school. For in these we undoubtedly find more of those "heart qualities" which are more essential towards the evolution of a nurse than are qualities of head. But the day in which we live demands smartness and mentality; it asks for a standard of brain athletics, and the nurse must needs give the world what it seeks. We are constantly told that nurses are becoming harder and more unsympathetic in manner, and that they have not the gentleness and pity and comforting presence of a time gone by. But the accusation is true of all women. We bicycle, we play hockey, we are smart and businesslike, and naturally we are losing our tender womanly ways. It is a stage of evolution we had to reach in order to acquire a different set of faculties, and, no doubt, we shall emerge well from the crisis and come out strengthened and developed. But transition stages are never particularly pleasant.

Having discussed the lofty ideals of our own and lay literature on what the nurse should be, it might, perhaps, be well to consider a more average standard to aim at.

It appears to me the best nurse is that woman whose maternal instincts are well developed—this being a chief reason why the modern woman is not essentially a good nurse-type. For the maternal instinct in the woman of to-day is distinctly in abeyance. The connection between mothering and nursing is very close, and undoubtedly the value of this old-fashioned quality gave rise to the conviction, now outlived, that no woman under fifty years of age was suited to be a professional nurse. New ideas on this point are in vogue —and rightly so—for modern nursing calls for plenty of healthy strength to go through its continuous and assiduous duties. It demands a clear brain, strong nerves, and young cheerfulness—but I pray you let these be combined with a large, kind motherliness! This was the saving grace of the older-fashioned nurse. This was her vantage ground.

The first axiom to be laid down is that nursing is not an intellectual pursuit. It calls for intelligence, but it is not *per se* suited to the intellectual woman, to whom High School teaching, literature, or mathematics would be a more congenial calling. Nursing is an art—not a science—and is best served by those with the artistic, sympathetic, and perceptive temperament; a combination of this disposition, with the practical qualities which training in hospital evolves produces the perfect—the ideal nurse. But unfortunately the common error of ranking intellect as above art has for the time being brought discredit on those more sympathetic and really womanly qualities of which the world at present stands badly in need. During several illnesses, which I rejoice to have had, for nothing helps better than our own illness to formulate an appreciation of the qualities best suited to the sick-room—I have been in the hands of the best and worst types of nurse, and their contrast affords a liberal education. Frankly, I must confess to have shivered under the cold glance of a critical scientific nurse, who promptly took my temperature to satisfy herself that the chilliness produced by her unsympathetic magnetism was not due to a rigor! She was a valuable object-lesson as she entered the room alert and smart; her training had been admirable, she had an excellent record of work done, and was taking her first private case. She wore a jangling chatelaine — cannot somebody in the interests of the sick produce a perfectly noiseless chatelaine?—and her starched gown rustled aggressively as she crossed the room. Glancing at the thermometer, before looking at the patient, she discovered the room was not theoretically warm enough, she therefore made up the fire, and did it Hospital fashion, with a muscular poke, and a mighty sound of falling cinders. In Hospitals it is quite impossible for quietness to prevail, for a ward must necessarily and naturally be subject to much passing to and fro, and the patients as a rule do not resent noise, having been long accustomed to it. The working classes live in public, in lodging houses, tenements, and noisy neighbourhoods, and it is very rare for any but the quite old to complain of a want of quiet in a ward. In less than half an hour I resigned myself to the fact that my room and I were in the hands of a "Reformer." Without question the spirit of reform is a stern necessity, it has done good service in the past for State and country. But the reformer himself is not a pleasant person, especially in a circumscribed sick-room. John Knox, no doubt, was a most estimable individual and a hard worker in a good cause. But the staunchest Protestant would hardly choose him as the type to soothe his sickness and pain, or smooth the pillows for the weary, for reformers are trying even to those in rude and ruddy health. But a reformer in the sick-room is distinctly harmful. A friend nursed by a woman of this type thus summed up his experiences: "I felt like a private on parade—my nurse standing for the captain of the squad.

### The Royal National Pension Fund for Nurses.

COMPETITORS FOR THE BUSINESS OF THE PENSION FUND.

The following remarks by Mr. King, the consulting actuary, at the annual general meeting, will be read with interest by all subscribers to the Fund :—

In answer to Mr. Burdett, who said : I beg leave to ask our actuary a question. It has been stated, Mr. King, that you did not personally work out our premium rates. Is there any foundation for this?

Mr. King replied : In reply I can only say that if anyone doubts that I gave personal attention to the preparation of the tables of rates, they have only to inspect the manuscript which is all in my handwriting. Not only did I consider on what principles the rates should be based, but I took the course very unusual with an actuary to make all the calculations myself. Not only so, but I continue to make the Royal National Pension Fund the first claim on my extra-official attention. Of course, I must give my first thoughts to the company to which I have the honour to be actuary, but next to that the Pension Fund has my first care, and I can assure you that a great deal of trouble is involved. A great many members appear not to know their own minds, and when they have taken out an annuity in one form they presently desire to change it to another. This necessitates intricate calculations, and a great deal of time is occupied in making them, and these I have always attended to myself. I think it very important to do this work, although it is very troublesome, because the Fund is intended to be for the benefit of the members, and their wishes should, as far as possible, be considered. Perhaps, when I am speaking, I may be permitted to say a few words regarding the competition to which the Fund is subjected. I do not think the Fund need fear competition. It is thoroughly sound financially, and it offers benefits to its members far beyond any other institution, and if taken on its merits it can fully hold its own. At the present moment there is a threat of competition from a source which may prove serious. An industrial company has prepared a scheme for nurses and others, which it proposes to push, and from the lowness of the rates that scheme is attractive. I have examined these rates and in my opinion they are actuarily unsound. In fact, it seems to me that the company in question is proposing to transact this business below cost price, and practically to make a present to its policy-holders of a certain part of the benefit. This, I may say, is not only in my opinion. It happens that only to-day I heard that another actuary had been consulted on the point, quite independently of this Fund or its management, and that he expressed precisely the same views. It may be asked why should nurses not accept such a present if they choose to do so. That is, of course, their affair. But I would submit that it is desirable to look such a gift horse in the mouth, because if a company deliberately transacts one branch of its business at a loss, it becomes a question whether that company is altogether the best to assure in. But even passing this point, I have no hesitation in saying that the Pension Fund need not fear such competition, because after all it is able to offer greater advantages. It must be remembered there are no shareholders in the Pension Fund, and there are no dividends to pay. Therefore, even supposing that we charge premiums beyond what are necessary for the contract benefits, any surplus premium charged will be returned as bonus, and the member will derive full benefit from every penny she pays. Not only so, but there is the donation bonus fund, from which very substantial additions will be made to the contract benefits. This will more than make up for the low rates charged by the competing office. Moreover, it must not be forgotten that all the arrangements of the Pension Fund are planned for the convenience of the members, and, therefore, it is not only the rate of premium that must be regarded. The exact description of the annuity must be considered ; whether, for instance, it is payable up to the day of death or not, and it will be found that on this point the Pension Fund is more favourable than the majority of its rivals. Also as to the payment of premiums, every facility is given to make this easy to the members, and if from any cause they are unable to pay these when due, they are assisted, and are not forced to terminate their contract. At all points their convenience and welfare are considered, and, in my opinion, it will be emphatically to their advantage to come to the Pension Fund rather than go elsewhere. (Hear, hear.)

### Victoria Commemoration Club.

We understand that, through Miss Thomson (the secretary), Sir Henry Irving kindly invited twelve members of the Victoria Commemoration Club to witness a performance of "Richard III." at the Lyceum. Mr. George Edwardes has promised Miss Thomson that members of this club will be allowed to book reserved seats at the Gaiety Theatre at half the usual price. They will, however, be required to show their ticket of membership when booking. It is exceedingly kind of Mr. Edwardes to have been so liberal, and we all know what a rest it is to a nurse to be able to go to a theatre comfortably.

On March 17th Mr. Charles Heath gave a most interesting lecture on the "Nursing of Cases of Throat, Nose, and Ear Diseases" to an appreciative audience. On March 23rd Dr. Drummond Robinson lectured on "Antiseptics in Midwifery Practice."

On March 30th, at 3 p.m., Dr. Ewen Maclean commences a course of six lectures on the nursing of gynæcological operations, including abdominal sections; and on the same date at 7 p.m. Miss Earle begins her course of lectures on "The Cooking of Dainty Dishes for the Sick Room." The lectures will be delivered weekly. Tickets for both courses may be obtained from the Secretary at 5s. each course to members, 7s. to non-members. Single lectures 1s. to members, 1s. 6d. to non-members.

### Presentations.

Bristol Hospital for Sick Children and Women.—Miss Combe, who has occupied the post of matron at this hospital for the last 15 years, having resigned in consequence of ill-health, the Committee of Management, through the President (Mr. Mark Whitwill, J.P.), on Saturday last presented her, at the close of the annual meeting, with a silver teapot, with suitable inscription, together with a purse containing bank notes for £55, with expressions of their deep regret on losing her. The staff nurses also gave Miss Combe a nicely-fitted leather dressing case.

### Novelties for Nurses.

BUTTER SCOTCH.

Messrs. Parkinson have sent us samples of their celebrated Royal Doncaster Butter Scotch. This sweetmeat has maintained a well deserved popularity so long that there is little left to be said in its favour. We can testify to the fact that its excellence is maintained, and that it is in every way a first-class sweetmeat made of wholesome ingredients and of agreeable flavour.

### Notes from South Africa.

From a Correspondent.

It is said, with much truth, that the sick room affords excellent studies of character; pain and weakness break down the conventional barriers behind which we usually hide ourselves, and the sick man shows himself as he really is. This is, to a certain extent, the case with uncivilised as well as civilised people; for although uncivilised people do not wear the same mask of conventionality that we do, still sickness brings out traits in their character which do not appear in health.

My own observations, made amongst semi-civilised Kaffirs and Hottentots, have led me to the following conclusions: They will make a great to-do about a very small ailment, such as a tooth-ache or a head-ache, but it is generally for the sake of getting something by it, or shirking work; on the other hand, they will sometimes show great endurance, sitting up and walking about when they are almost dying, and not shrinking from the severest pain when they mutilate themselves for a religious or other purpose. They still prefer their own Kaffir doctors, and their own original and sometimes disgusting remedies, to a white doctor, and the resources of modern medical science; and in regard to sick-nursing, they are, almost without exception, totally and hopelessly ignorant.

In describing an illness a Kaffir will exaggerate *ad libitum*, so that a person who has very little the matter with him is represented as being at the point of death. If you try to find out what complaint he is suffering from, you will probably be baffled by the extreme vagueness and obscurity of the description. A favourite expression is that "his whole body is sore," but, as that is a symptom common to many complaints, it will not be of much assistance to the inquirer.

They are ready to take any remedies that are given to them, and will often come and ask for vinegar for a cough, or paraffin to rub a sore leg, &c. They can by no means be trusted, however, to administer medicine at regular intervals, or in proper quantities; they like to give it all at once in a tremendous dose; it is less trouble, and seems more likely to do good that way.

If a native is taken suddenly ill, great alarm is manifested by his friends; they all crowd round him, excluding every particle of fresh air, and wail and lament loudly, wringing their hands, but not attempting to do anything for him. On the other hand, if a native has a long, lingering illness, he receives no sympathy and very little care from his own people. They are very unkind to their sick, as far as I have seen, often neglecting to feed them, and never even thinking of making their beds or changing their clothes. As they lie down so they remain, until they either die or, through the healing influences of Nature, recover and get up again. It is impossible to imagine a more pitiable object than a sick person in a Kaffir hut. I went once to see a Kaffir woman who had had influenza, and lay for weeks in a state of extreme weakness. The hut was, as usual, small and low, choked with wood smoke, and full of pots and pans and general litter. The poor woman lay on an old mattress, on the ground, in all her clothes, which she had never changed, and with a very dirty blanket over her. It was summer, and the flies swarmed in the hut. They crawled all over the sick woman's face, and she was too weak to lift her hand to brush them away. Her daughter, a tall, strong young woman, stood looking at her, never even offering to brush the flies from her face, never thinking of washing out a blanket or a gown for her, simply leaving her to lie there in the utmost misery and discomfort. At night a whole crowd of children, crying babies, dogs, and hens would come to sleep in that same hut, the atmosphere of which was already stifling. We brought her food—tapioca, ready cooked—but we knew that unless we stayed to feed her with it the other natives would probably eat it up. How do these people ever recover from sickness? That woman recovered—but how?

I knew one old woman who suffered from attacks of bronchitis every winter. It was the hardest work in the world to persuade that old woman to lie in bed. "No," she used to say, "if I once go to bed I shall never get up again." There was some truth in what she said, for it does seem to me that if a native is ill enough to take to his bed he seldom does get up again: they seem to think that then it is all over with them, and perhaps that is one reason why they do not recover as a rule.

The natives are very fond of their children, and are more attentive to them when they are sick than to adults or old people. Yet they do not show the slightest commonsense in the management of their babies, and the most they can do for a sick child is to howl and weep over it. A nurse who tries to work amongst the natives will have a great deal to contend with. Their prejudices and their superstitions constantly interfere with any sensible treatment, and their homes are, to our ideas, absolutely without comfort. You can never be sure that they take any food or any medicine that you do not give them with your own hands; and, worse still, while they take the medicine that you give them, they will often, at the same time, be secretly taking medicines given them by the Kaffir doctor, so that you never know where you are with them.

Civilisation has yet much to do in this direction among the native tribes of South Africa. Yet in the hospitals the natives are the best patients. They are more accustomed to do what they are told, so they give less trouble; and they have fewer fads and fancies than white people, not having yet attained the same development of "nerves." Hospital nurses generally prefer the native wards, I am told.

And, after all, the worst that can be said is this, the natives are very emotional, are lacking in self-control, and, in sickness, are ignorant and helpless; but how many white people, after centuries of civilisation, are the same! What education can do for the one it will, no doubt, in time, accomplish for the other.

### Minor Appointments.

City of London Union Infirmary.—Miss S. A. E. Richmond has been appointed Superintendent Night Nurse at this infirmary. Miss Richmond was trained at St. Thomas's Hospital, subsequently holding the post of staff nurse at the New Hospital for Women for three years, at Charing Cross Hospital for nearly two years, and that of superintendent night nurse at the Paddington Infirmary for two years.

Children's Hospital, Sheffield Union.—Miss Edyth M. Davis has been appointed Charge Nurse at this institution. Miss Davis was trained at the West Ham Hospital, Stratford, E., where she afterwards became sister of the women's and children's wards, and was subsequently appointed sister of surgical ward and operating theatre at the Belfast Hospital for Children.

Milton Hospital, Portsmouth.—Miss Alice Gough has been appointed Staff Nurse at this hospital. Miss Gough trained at the South-Western (M.A.B.) Hospital, and has held appointments at the South-Western Hospital, at Gore Farm, and at Bristol General Hospital.

Felsted School Infirmary.—Miss Annie Proctor has been appointed Nurse at the infirmary of this school. Miss Proctor previously held an appointment at the Western Fever Hospital, Fulham.

Blything Union Infirmary.—Miss Alice Glanfield has been appointed Nurse at this infirmary. Miss Glanfield was trained at University College Hospital.

Miss Kendrick, whose appointment at the Derby County Asylum was noted a few weeks ago in this column, asks us to state that she gained experience as head nurse at the Dorset County Asylum.

### The Registration of Midwives.

### MEETING AT LONDON HOUSE.

A WELL-ATTENDED meeting was held last Thursday at London House, St. James's Square, in support of the Midwives Registration Bill, at which Mrs. CREIGHTON presided. The promoters of the meeting had every reason to congratulate themselves on its unqualified success. There was an excellent array of speakers, and the audience was quite enthusiastic in its support of the various resolutions brought forward.

Mrs. CREIGHTON briefly summed up the arguments in favour of the Bill, and Dr. CHAMPNEYS, in a convincing speech, proposed the resolution: "That this meeting is convinced of the urgent necessity in the interests of the community of some measure for the registration and supervision of midwives." It was computed, he said, that there were from 10,000 to 15,000 midwives at work in England and Wales, attending nearly half a million women yearly. He spoke of the numbers of victims to puerperal fever—deaths (the saddest of all, perhaps, in the community) which could be prevented, and were due to the unsatisfactory women who attended confinements. There were only three courses to be taken—to abolish midwives, to regulate them, or to leave them alone.

Dr. ANNIE MCCALL seconded the motion, and emphasised the fact that the regulation of midwives was a woman's question, and mainly a poor woman's question. She pointed out the undoubted fact that many women preferred to be attended in their confinements by women, that not only could the midwife give her services for far less than the doctor, but she also acted as nurse for the baby, and helped in little domestic ways as a woman alone could do. Speaking from her experience in training women students, and in her work at the Clapham Maternity Hospital, she thought three, four, or six months' training was sufficient to teach the average woman when to send for a doctor, and to render her a safe person for a poor woman to employ. Dr. McCall concluded by remarking that England was the only European country which had not legislated on this matter.

Dr. GEORGE BROWN opposed the resolution, which was carried with three dissentients. Mr. SCHWANN, M.P., moved a resolution approving of the Bill and urging its acceptance by the Government, without whose help he doubted the possibility of getting it through Parliament this session. He explained that the details of the training which would be considered necessary for qualified midwives would be left to the Midwives' Board, upon which there would be representatives of the General Medical Council. Dr. Cullingworth seconded the motion, and Mrs. Charles McLaren made a bright little speech urging those present to interest themselves and their friends in the question.

Mrs. SOMERVILLE, who was very imperfectly heard at the end of the room, was understood to object to the Bill. Sir Frederick Fitz Wygram suggested the formation of a company instead of attempting to pass the Bill through Parliament (of which result he seemed to take a despairing view), a proposal which brought a spirited reply from Miss Wilson, president of the Association for Promoting Compulsory Registration of Midwives, who declared, amidst applause, that such a suggestion was absolutely futile, that nothing but a Bill would meet the question, and that until such a Bill was passed they would one and all have a heavy weight on their consciences. The resolution was carried almost unanimously, and a third motion "that copies of the resolutions passed should be sent to the Lord President of the Council and the Home Secretary" was proposed by Mr. H. Johnstone in an excellent speech, and duly seconded and carried.

A cordial vote of thanks was passed to Mrs. Creighton for presiding.

### Our American Letter.

(COMMUNICATED.)

THE great event in the American nursing world last month was the Fourth Annual Convention of Superintendents of Training Schools for Nurses which met in Baltimore. It was an eminently successful meeting, and very well attended. Several pleasant social gatherings took place at the Johns Hopkins Hospital and elsewhere, the meetings being held in the Donovan Room of the University. The second meeting of the convention first called a year ago by the Society of Superintendents to organise an American association of graduate nurses was held immediately following the Superintendents' Convention in the Nurses' Home of the Johns Hopkins Hospital.

A pleasant gathering took place lately at the New Orleans Charity Hospital Training School for Nurses, when numbers of past graduate nurses attended a banquet and a discussion, presided over by Mrs. Agnes O'Donnell, the superintendent. The occasion was taken to form an alumnæ association, a move greeted with much enthusiasm, and a committee and officers were duly appointed.

A Training School for Nurses, which has been long wanted, has been started in connection with the Albany City Hospital. A three years' course of instruction has been adopted, and Miss Emily MacDonnell, of the Johns Hopkins Hospital, Baltimore, and the City Hospital, Kingston, Ont., has been appointed Superintendent of Nurses. At the Delaware Hospital a similar step has just been taken, and a Training School for Nurses established, the course to extend over three years.

The need for a proper Nurses' Home in connection with the Hospital of the Protestant Episcopal Church in Philadelphia is forcing itself upon the attention of the board of managers, and a committee has now been appointed to see to the preparation of plans for a separate building to accommodate some eighty pupil nurses. The home is expected to cost, when completely fitted, about 100,000 dols.

### Everybody's Opinion.

[Correspondence on all subjects is invited, but we cannot in any way be responsible for the opinions expressed by our correspondents. No communication can be entertained if the name and address of the correspondent is not given, or unless one side of the paper only is written on.]

### CYCLING FOR NURSES.

NURSE S. writes: In answer to the district nurse's letter in last week's HOSPITAL, I would not advise a district nurse riding a bicycle with her apron and no cloak on. It would be likely to bring a nurse into ridicule, especially in the country. Such is my experience, and I have had a country district. I would recommend a uniform cloak with a cape which would allow of free movement of the arms. At the Nursing Exhibition held in London last summer, Messrs. Debenham and Freebody sent a model of a district nurse dressed as I have described.

### NURSES' ALMSHOUSES.

"A TRAINED NURSE" writes: I have read your paper for some years now, having been a nurse for nearly ten, and began to like it when I was a probationer at Guy's, where they kindly provided THE HOSPITAL for us. I have always found the paper useful in many ways, but have never ventured to write to you before; but I see all kinds of things written about in "Everybody's" corner, and wondered if you thought this idea that has suggested itself to me would be worthy of a place there. Why do not nurses celebrate this special year by contributing together for either almshouses or one large house divided off, to be in different parts of the country, to which nurses, when they are either too old for work or incapable by ill-health, could retire? The Pension Fund is, of course, a splendid thing for nurses; but if they are without relations or friends to live with, it would not be sufficient to keep them properly. Again, some nurses have home relations to keep now, and so are unable to join the Pension Fund. If all nurses contributed now it could soon be done, with annual subscriptions to keep them up. Open to all kinds of nurses. I would myself willingly pay £5 5s. a year towards such a good object while I am able to. At present I am always very well and busy, 36 years old; but I hope to work for many more years yet. I was six years at Guy's, also hold the L.O.S. certificate, and am a masseuse. I read once of a bed being kept for a sick nurse at the Brassey Home of Rest, and suppose it is still kept on. But I think for nurses to be together in their old age, one helping the other as they do in the beginning at the hospital, is something to look forward to, for some of us must outlive our own friends and relations, and a solitary old age is not cheerful to think about.

### A "UNIFORM" QUESTION.

A DISTRICT NURSE writes: I should so much like the opinion of readers of THE HOSPITAL upon a little matter with regard to the wearing of uniform when not on duty. I am a district nurse, and for the sake both of economy and convenience I should like to wear uniform altogether both when on and off duty. Would there be any objection, for instance, when invited out in the evening, when other guests would be in evening or full dress, against my wearing a navy blue silk or alpaca, plainly made, after the same style as my ordinary serge, with nurses' collar and cuffs? If I were private nursing I should have no difficulty in the matter, as in an official capacity I should feel quite comfortable in the house of my patient or their friends, but in the case of a district nurse it seems to me a question whether it would be good form to do this, and as I should not wish to show any disrespect to any friends for the sake of my own comfort, I should like to know what would be the correct thing to do.

*** We have received several queries on this subject, and it would be very helpful to our correspondents if nurses, district or otherwise, would send us their views on this matter. Nurses will all appreciate the "convenience" side of the question, but there are other points to be considered, and we shall be very glad to hear what our readers' opinions are on the matter.—ED. *T. H.*

### Wants and Workers.

*Bath Chairs.*—Nurse Kennedy, Enfield parish nurse, would be very grateful for a Bath chair for the use of the sick poor, or nurse would pay a trifle for light secondhand one.—Nurse Underwood, district nurse at Hartfield, near Tunbridge Wells, asks "if some kind friend would give a bath chair for some old people crippled up with rheumatism, to take them out?" The village is a poor one, with no rich people to help in providing this comfort. Nurse Underwood would gladly pay carriage. Her address is "Green Cottages," Hartfield.

*Home Wanted.*—The matron of the Children's Cottage Convalescent Hospital, Wokefield, Mortimer, Berks, writes: Can you or any of your correspondents tell me of a home where a little girl of four, with curvature of spine could be received free of cost? The child is quite able to walk about, but is too delicate to be sent to her home, as her father is in consumption and her mother earns 2s. a-week at laundry work, and there are three other young children dependent on them. We have consulted "Burdett's Hospital Annual," and find that at the homes mentioned there the children eligible must be either destitute or orphans.

### Notes and Queries.

*Nursing.*

(182) Please tell me (1) Where can I obtain particulars of the Royal British Nurses' Association? (2) How could I best obtain a post in a good private nursing association in London? Is there a list of such institutions obtainable?—*Anxious.*

(1) Apply to the secretary of the association, 17, Old Cavendish Street, W. (2) You will find the principal private nursing institutions in London given in "Hospitals and Charities" (Scientific Press, 28 and 29, Southampton Street, Strand, W.C.) The most successful association in London is the Nurses' Co-operation, 8, New Cavendish Street, W., managed by nurses themselves. Write to the secretary for the rules. Nurses are elected on the staff in May and November each year.

*Army Nursing Reserve.*

(183) I was interested in reading about the "Army Nursing Reserve" in a recent number of THE HOSPITAL. Please tell me where full particulars may be obtained?—*Heathcote.*

Apply to the War Office for particulars.

*Private Nursing.*

(184) I am anxious to take up private nursing, and should feel grateful if you could tell me anything about the Nightingale Home for Trained Nurses at Southsea.—*A Five Years' Nurse.*

Do you mean the "Nightingale House for Trained Nurses and Private Patients," Osborne Road, Southsea? Miss Tillett is the Lady Superintendent, and you had better write to her for full particulars.

*Two Years' Training.*

(185) Please advise me. I am a probationer at a women's hospital, where my term of two years will be up in September, and I must then train at a general hospital. I wish to enter for two years only, which my present matron says should be quite sufficient, as she says that in many cases general hospitals send their third year nurses out to private cases. Can you tell me of any institutions where a two years' course may be had? I suppose it pays hospitals better to keep nurses for three years, and so it is useless to expect the authorities to make any alteration in their rules. I have enjoyed your articles on "Nurses in 1896," and wish they could have been extended to good provincial hospitals.—*St. George.*

You are quite right. Two years' general training should be quite sufficient for yourself, but it does "pay hospitals better" to bind their nurses for longer periods, and so most of them have adopted a three years' course, at least in London. There are still good provincial hospitals where excellent training may be had, and where probationers can enter for two years. Apply to Miss Carvosso at the Derbyshire Royal Infirmary. Unless quite recently altered, a two years' course of training is given there. Write also to Miss Watt at the Radcliffe Infirmary, Oxford, and Miss Johnstone at the Salisbury Infirmary. At Macclesfield Hospital and Sheffield general Infirmary a two years' course, we believe, is given. It would be of no use to ask for exceptions to be made in the rules. We hope in the future to treat of the training, &c., at provincial hospitals.

*Six Months' Training.*

(186) Could I get six months' training in a general children's hospital somewhere near Warwick, where a small salary and uniform would be given?—*Children's Nurse.*

You can only get short training by paying for it. Why not apply to the Matron of either the Derbyshire Hospital for Sick Children, North Street, Duffield Road, Derby, or the Birmingham Hospital for Sick Children (Broad Street), for terms of training, and if you cannot afford to pay, go in for the two years' course.

*Bandaging.*

(187) Where could I get some instruction in surgical bandaging?—*Mental Nurse.*

Write to the Secretary of the Trained Nurses' Club, 12, Buckingham Street, Strand, enclosing stamped envelope for reply.

*An Extraordinary Notion.*

(188) Nurse Dorothy Smith asks if it is true that people "during life sell their bodies to the hospital medical schools to be claimed after death for dissection?"

We believe some people have been anxious thus to dispose of themselves, but Nurse Dorothy Smith surely cannot seriously imagine that such an arrangement would be entered into by the authorities of "hospital medical schools"!

### Where to Go.

GROSVENOR HOUSE.—The twenty-first annual meeting of the Metropolitan and National Nursing Association will be held at Grosvenor House on Friday, April 2nd, at 3 p.m.

CRYSTAL PALACE.—A fancy dress ball is to be held on April 28th at the Crystal Palace in aid of the Nursing Division of the Soldiers and Sailors' Families' Association, of which particulars may be had from Lady Methuen, 32, Cadogan Square, or Mrs. H. C. Hamilton, hon. secretary, Aros House, Norwood. The ball is under the patronage of the Duke and Duchess of Connaught, Princess Christian, and Princess Louise, Marchioness of Lorne.

